# R-loops as a trigger for intra- and extrachromosomal DNA amplification in cancer

**DOI:** 10.3389/fcell.2025.1647255

**Published:** 2025-12-03

**Authors:** Tatyana F. Kovalenko, Amal Abdurazakov, Nadezhda V. Antipova, Mikhail I. Shakhparonov, Marat S. Pavlyukov

**Affiliations:** 1 Shemyakin-Ovchinnikov Institute of Bioorganic Chemistry, Moscow, Russia; 2 Higher School of Economics University, Moscow, Russia

**Keywords:** R-loops, double strand breaks, replication stress, DNA amplification, ecDNA

## Abstract

R-loops consist of double-stranded DNA-RNA hybrids and a complementary DNA strand that is displaced from the duplex. R-loops play important role in numerous normal physiological processes, including DNA methylation, chromatin remodeling, RNA editing, replication, DNA repair, immunoglobulin class switching, and chromosome segregation during cell division. However, excessive or untimely formation of R-loops can lead to replicative collapse and subsequent DNA damage, resulting in genomic instability. One type of genomic rearrangements that is strongly associated with cancer malignancy is the extrachromosomal amplification of genes on circular DNA molecules (ecDNA). These molecules are relieved of hereditary constraints and conventional segregation laws and can endow cancer cells with the ability to rapidly change their genome, thereby accelerating tumor evolution and the development of therapy resistance. Multiple lines of evidence indicate that upregulated transcription of a gene can increase its susceptibility to amplification. Although the mechanisms underlying these processes are not yet fully understood, R-loops may play an important role in initiating gene amplification. In this review, we highlight the role of R-loops in replicative collapse, double-strand breaks, and DNA damage repair. We also provide examples of gene amplifications that is known to be induced by R-loops. Finally, we discuss amplification mechanisms in which involvement of R-loops has not yet been demonstrated, but appears highly likely.

## 1 Introduction

R-loop is a structure in which an RNA molecule hybridizes with one strand of double stranded DNA and the other strand is displaced from the helix, forming a single-stranded DNA (ssDNA) region (Palancade and Pothstain, 2021). R-loops are widespread in living organisms. Short and transient R-loops frequently arise during transcription due to the annealing of newly synthesized RNA transcripts on the unwound DNA template. However, in some cases, more stable and extended R-loops are formed and can persist even after RNA polymerase has dissociated from the DNA (Castillo-Guzman et al., 2021). In addition to R-loops that are formed cotranscriptionally *in cis*, posttranscriptional *in trans* formation of these structures have also been reported. In such cases, RNA molecules synthesized in one genomic region hybridize with DNA sequences at another region (Feretzaki et al., 2020).

**FIGURE 1:**
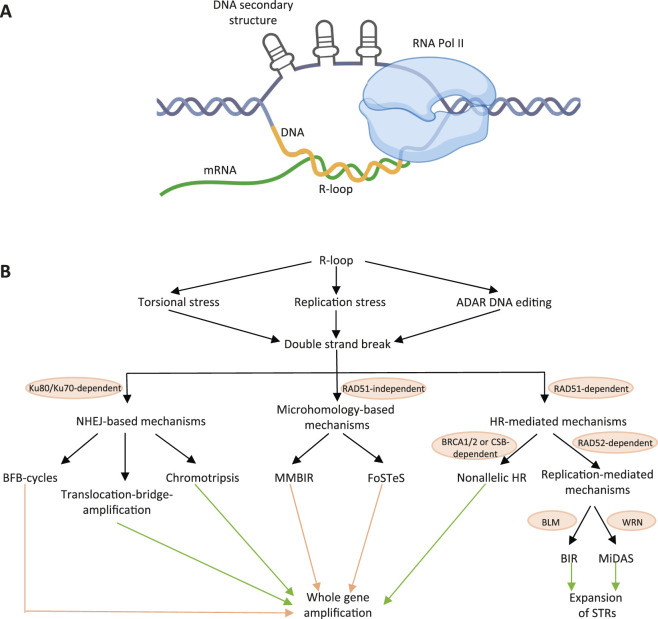
Interconnection of R-loops with DNA amplification. **(A)** Schematic representation of the R-loop structure. DNA secondary structures that stabilize the R-loop are shown on the single-stranded DNA region. **(B)** Diagram illustrating mechanisms linking R-loops to the amplification of DNA regions. Green arrows indicate processes in which the involvement of R-loops has been experimentally demonstrated. Orange arrows indicate processes in which R-loop participation has not yet been confirmed but considered highly likely. Schema was created using BioRender. Abdurazakov, A. (2025).

Certain DNA regions are particularly prone to forming stable R-loops. These include GC-rich sequences, often located near Transcription Start Sites (TSS) (Dumelie and Jaffrey, 2017), as well as repetitive genomic elements such as ribosomal RNA genes, centromeric and telomeric repeats, and various types of short tandem repeats (Abraham et al., 2020; Kabeche et al., 2018; Groh et al., 2014). If an RNA transcript hybridizes with a DNA template rich in cytosines, the displaced G-rich complementary DNA strand can form various secondary structures such as G-quadruplexes (Figure 1A). They stabilize R-loops by preventing DNA reannealing (Groh et al., 2014; Loomis et al., 2014). It has also been shown that negative DNA supercoiling enhances R-loop stability (Manzo et al., 2018).

R-loops are involved in numerous normal physiological processes: they act as regulators of DNA methylation (Arab et al., 2019), chromatin remodeling (Chen et al., 2015), RNA editing (Cui et al., 2022), transcription (Beckedorff et al., 2013; Tan-Wong et al., 2019), DNA replication (Wiedemann et al., 2016), and DNA repair (Teng et al., 2018; Yang et al., 2023). In addition, R-loops promote chromosome segregation during cell division (Kabeche et al., 2018) and contribute to genomic rearrangements that mediate immunoglobulin class switching (Yu et al., 2003; Wiedemann et al., 2016). However, excessive or untimely formation of R-loops can lead to DNA damage and genomic instability associated with cancer (Costantino and Koshland, 2018). These processes are initiated by Double-Strand Breaks (DSBs) which arise due to R-loops-induced replication stress, torsional stress or ADAR-dependent DNA editing (see Section 2).

There are several mechanisms that help cell to remove R-loops. These include unwinding of DNA-RNA hybrids by specific helicases (SIRT7, DDX21, DDX41; SETX) (Song et al., 2017; Mosler et al., 2021; Gatti et al., 2023), cleavage of RNA within DNA-RNA duplexes by RNase H1 or RNase H2 (Shen et al., 2017; Kojima et al., 2018), relaxation of negative supercoiling by topoisomerase I (Top I) (Manzo et al., 2018), and chromatin remodeling by proteins such as ATRX (Nguyen et al., 2017). Reduction of R-loops levels is also facilitated by nuclear export of RNA through the THO/TREX complex (Hamperl and Cimprich, 2014), as well as by the splicing of pre-mRNA molecules (Li and Manley, 2005). However, excessive formation of R-loops may hinder their timely removal by these “safe” mechanisms. In such case, R-loops processing can be carried out by nucleases (XPF, XPG), which cleave the DNA component of R-loop (Sollier et al., 2014) and trigger a cascade of DNA damage response reactions, that may provoke genome instability–an important hallmark of cancer (Sollier et al., 2014; Stork et al., 2016).

Genome instability can lead to alterations in the copy number of specific chromosomal regions, known as Copy Number Variations (CNVs) (reviewed in Hastings et al., 2009). One type of CNVs is amplification, which can occur both under normal physiological conditions (e.g., during specific developmental stages (Sinclair and Guarente, 1997; Gall et al., 2004)) and in cancers, where it contributes to tumor malignancy (Hastings et al., 2009). Thus, amplification of various oncogenes, including *C-MYC*, *MYCN*, *JUN*, *EGFR*, *BRAF* and *CDK4* is strongly associated with cancer progression and the development of therapy resistance across multiple types of solid tumors (reviewed in Matsui et al., 2013). Therefore, identifying the molecular mechanisms that trigger gene amplification is essential for the development of novel anticancer therapies.

In this review, we focus on the role of R-loops in initiation of various types of DNA amplification (Figure 1B). First, we describe the role of R-loops in the development of replicative stress, the formation of DSBs, as well as the implication of R-loops in DNA repair processes that may lead to the amplification of various genomic regions. Next, we provide description of DNA amplification types, including forms of extrachromosomal DNA amplification (microDNAs, telomeric circles, small polydisperse DNA, and ecDNAs). We then summarize the currently known mechanisms of intra- and extrachromosomal DNA amplification initiated by DSBs: mechanisms based on homologous recombination (non-allelic homologous recombination, BIR, MiDAS), microhomology (MMBIR, FoSTeS) and non-homologous end-joining (breakage-fusion-bridge cycles, translocation-bridge-amplification, chromothripsis). Finally, we present evidence supporting the role of R-loops in initiating numerous gene amplification pathways and discuss amplification mechanisms in which R-loop involvement has not yet been demonstrated but appears highly likely based on indirect evidence.

## 2 R-loops as sources and consequences of double-strand breaks

The formation of stable DNA-RNA hybrids can lead to the development of replication stress (Gan et al., 2011). The term “replication stress” refers to a blockage in the progression of DNA polymerase during replication. If the cell removes such obstacle in a timely manner, the replisome remains intact and DNA synthesis resumes. However, if the replication block persists, the replication machinery dissociates from the DNA template, leading to replicative collapse (Macheret and Halazonetis, 2015). Stable DNA-RNA hybrids are among the major inducers of replicative collapse. Various mechanisms exist to eliminate R-loops and resolve replicative collapses, including the removal of DNA fragments that forms RNA-DNA duplexes. This process is carried out by the XPF and XPG endonucleases of the nucleotide excision repair (NER) pathway and leads to the formation of DSBs. There are two types NER mechanism in the cell: global genome NER (GG-NER) and Transcription-Coupled NER (TC-NER), which differ in their initiation mechanisms. GG-NER is activated by the formation of DNA cross-links and nucleotide adducts (Petruseva et al., 2014), whereas TC-NER is triggered by RNA-polymerase stalling, which can occur due to R-loops, and involves proteins such as CSA and CSB (Sollier et al., 2014; Stork et al., 2016).

Multiple studies have shown that the DNA chain in a DNA-RNA hybrid can be edited by ADAR enzymes (adenine deaminases), which normally act on RNA in RNA-RNA hybrids (Tang et al., 2022). As a result, deoxyadenosine (dA) is converted into deoxyinosine (dI). Next, an abasic site is generated by MPG (N-methyl-purine DNA glycosylase). This site is then cleaved by APE1, a nuclease of the Base Excision Repair pathway (BER), leading to the formation of a Single-Strand Break (SSB), which can be converted into a DSB upon subsequent DNA replication (Tang et al., 2022).

R-loops can also induce DSBs through mechanisms independent of DNA replication (Cristini et al., 2019; Hidmi et al., 2024). Cristini et al. demonstrated that DSBs can arise in highly transcribed genes as a result of two SSBs occurring on complimentary DNA strands within the transcription bubble. One SSB is generated by topoisomerase I (Top1), which normally eliminates torsional stress during transcription, while the opposing DNA strand acquires an SSB due to R-loop processing by endonucleases such as XPF, XPG, FEN1, or MRE11 (Cristini et al., 2019). Two closely spaced SSBs on complementary strands of unwound DNA lead to DSB formation.

Importantly, R-loops are not only a source, but also a consequence of DSBs. Several studies have shown that DSBs in actively transcribed genes promote R-loops formation. Thus, Cohen et al. demonstrated that DSBs induced by the restriction enzyme AsiSI promote the formation of R-loops near DNA damage sites (Cohen et al., 2018). Similary, Teng and colleagues showed that DNA damage, induced by Reactive Oxygen Species (ROS), initiate R-loop formation in actively transcribed genomic regions (Teng et al., 2018). In these cases, R-loops are formed by transcripts that have been already synthesized (or were in the process of synthesis) at the time of DNA damage. However, there are also examples where DSBs initiate *de novo* RNA synthesis. Several studies have shown that DNA ends generated due to DSB can recruit RNA polymerase II, thus functioning alike “promoter”. Transcription at these sites leads to the formation of damage-induced long non-coding RNAs (dilncRNAs) (Michelini et al., 2017), which can form R-loops (Shaw and Gullerova, 2021) and may subsequently serve as precursors for small non-coding RNAs involved in DSB repair.

## 3 R-loops and double-strand break repair

Regardless of whether R-loops are the cause or consequence of DSBs, these structures are closely linked to the DNA repair machinery. Double-strand break repair proceeds primarily through two pathways: Homologous Recombination (HR) and Non-Homologous End Joining (NHEJ) (reviewed So et al., 2017). The role of R-loops in HR has been convincingly demonstrated by several studies (Teng et al., 2018; Yang et al., 2023) and there are also evidences suggesting a link between R-loops and NHEJ (Yang et al., 2023).

HR occurs during the S and G2 phases of the cell cycle and requires regions of homology. This process involves multiple factors, including the MRN complex, RPA protein, RAD51, RAD52, BRCA1, and BRCA2. R-loops have been shown to recruit BRCA1/2 and RAD52 proteins, while the RPA protein, which binds to ssDNA, is typically present on the DNA strand displaced from the duplex by the R-loop (Teng et al., 2018). In addition, R-loops can promote HR via a BRCA1/2 independent mechanism. This pathway requires recruitment of the CSB protein to the DNA-RNA hybrid which facilitates localization of the RAD51 recombinase at the R-loop site. Activation of this mechanism has been observed during R-loops formation initiated by ROS-induced DNA damage (see section 2) (Teng et al., 2018).

There is also intriguing evidence suggesting that modifications of RNA molecules forming DNA-RNA hybrids can determine the choice of DNA repair pathway. Thus, it has been shown that the presence of a methyl group at 5-position of cytosine RNA molecule (m^5^C) promotes DSBs repair via HR, whereas the absence of this modification favors alternative NHEJ (alt-NHEJ) pathway, which depends on microhomology regions (Yang et al., 2023).

In summary, R-loops are intimately linked to HR and NHEJ-mediated DNA repair. While both HR and NHEJ have been shown to promote intra- and extrachromosomal amplification of genomic regions. Therefore, in the following section, we will discuss the known mechanisms of R-loop-associated genome rearrangements, with a particular focus on DNA amplification.

## 4 Types of DNA amplification

There are multiple criteria by which amplification of chromosome regions can be classified: the number of copies of the genomic fragment, the size of the amplified region, and its localization. The number of extra copies can vary from one (duplications) to several thousand, as observed in the case of the short repeating motifs expansion (Malik et al., 2021; Bagshaw, 2017). The size of the amplified region can range from a few nucleotides, to multiple genes or even entire chromosomes (reviewed by Pös et al., 2021). Finally, the amplified region may be localized either within the chromosome or in extrachromosomal DNA elements.

Extrachromosomal structures are mainly represented by extrachromosomal circular DNAs (eccDNAs) which vary greatly in size and mechanisms of formation (reviewed in Liao et al., 2020; Ling et al., 2021). eccDNAs include microDNAs (100–400 bp), telomeric circles (T-circles; ∼700 bp), small polydisperse DNAs (spcDNAs; ∼ 1–10 kb), and the largest circular DNA molecules, ecDNAs, which can reach up to 3 Mb in size. Most eccDNAs are capable of autonomous replication, which may promote further increase in the copy number of the corresponding genomic region (Liao et al., 2020; Ling et al., 2021). MicroDNAs and spcDNAs are detected in both cancer cells and normal somatic cells of adult organisms (Dillon et al., 2015; Møller et al., 2018). T-circles are found in embryonic tissues and in cancer cells that utilize the alternative telomere lengthening (ALT) mechanism (Hou et al., 2022). ALT enables telomere elongation without the involvement of telomerase. Briefly, this process is based on homologous recombination, in which the telomeres of sister chromatids serve as template for telomeric repeat synthesis. (Min et al., 2017) (see Section 6.2). ecDNAs are detected exclusively in cancer cells (Liao et al., 2020; Ling et al., 2021).

MicroDNAs contain unique genomic sequences flanked by short direct repeats. Their mechanisms of origin are not yet fully understood, and they are predominantly formed from GC-rich, actively transcribed regions of the genome (Dillon et al., 2015). T-circles contain only telomeric repeat sequence and arise either through intramolecular HR between chromosomal telomeric repeats (see section 5) or due to excision of telomeric loops (Liao et al., 2020). spcDNAs are enriched in repetitive genomic elements but may also contain unique sequences. These molecules can be formed via intramolecular HR or end joining at microhomology regions (Liao et al., 2020; Møller et al., 2018). Finally, ecDNAs comprise entire genes or even gene clusters, along with regulatory elements such as enhancers and super-enhancers (Yang et al., 2025). Current evidence suggests that these molecules can arise through the Breakage–Fusion–Bridge (BFB)- cycles or chromothripsis (Liao et al., 2020; Ling et al., 2021) (see section 5).

Importantly, circular DNAs lack centromeres. As a result, they are unevenly distributed between daughter cells during mitosis, leading to large variations in ecDNA copy number within a cell population. In the case of ecDNAs, this uneven segregation contributes substantially to the development of intratumoral heterogeneity and, as a result, may promotes the rapid adaptation of cancer cells to various therapies (Yang et al., 2025).

Similar to eccDNAs, DNA fragments undergoing intrachromosomal amplification also vary widely in size. For example, amplification of short tandem repeats can involve regions of only a few nucleotides long (Bagshaw, 2017; Malik et al., 2021), whereas extended amplicons spanning several megabases have also been described (Morales et al., 2009). Notably, numerous studies indicate that intra- and extrachromosomal amplification are capable of mutual transition. DSBs within regions of intrachromosomal amplification may lead to the formation of eccDNAs, while circular DNAs can reintegrate into the genome, generating amplified DNA in the chromosome (Morales et al., 2009; Song et al., 2022).

## 5 Mechanisms of R-loop-associated intra- and extrachromosomal amplification initiated by double-strand breaks

The mechanisms of intra- and extrachromosomal amplification have been described in numerous reviews (Hastings et al., 2009; Matsui et al., 2013; Liao et al., 2020; Ilić et al., 2022). Some of these pathways–such as DNA polymerase slippage or overreplication - are based on disturbances in the replication machinery that are not associated with R-loops. In this section, we describe mechanisms that may result from the formation of stable DNA–RNA hybrids: HR-related pathways (non-allelic HR, Break-Induced Replication (BIR), Mitotic DNA Synthesis (MiDAS)), microhomology-based mechanisms (Microhomology-Mediated BIR (MMBIR), fork stalling and template switching (FoSTeS)) and NHEJ-based pathways (BFB-cycles, the translocation–bridge amplification model, and chromothripsis).

### 5.1 Amplification mechanisms based on homologous recombination

**FIGURE 2:**
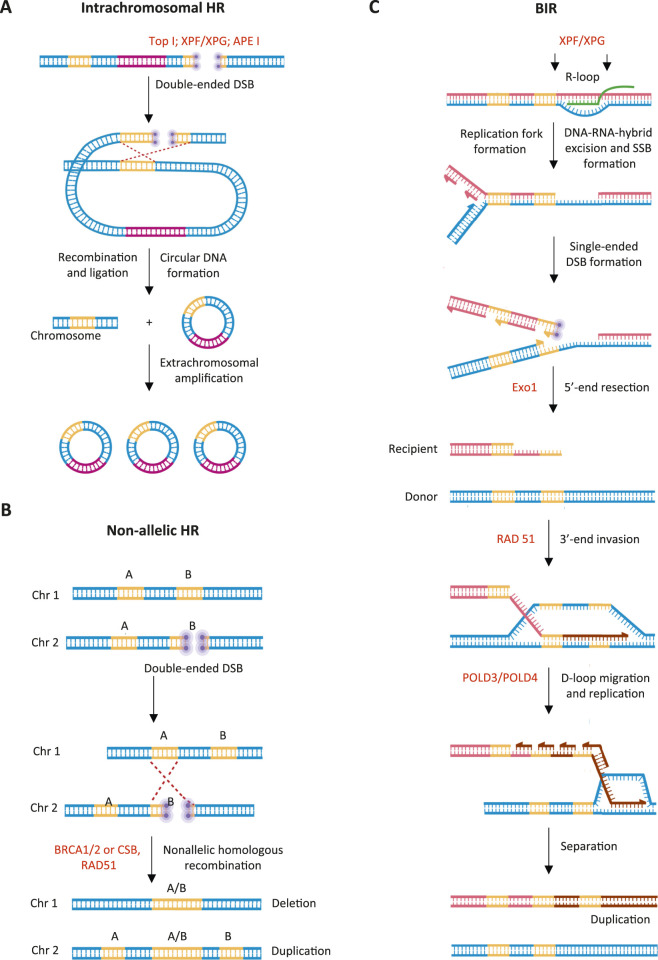
HR-associated amplification mechanisms. **(A)** Intrachromosomal recombination and formation of autonomously replicating circular DNA. **(B)** Non-allelic recombination between homologous chromosomes, leading to intrachromosomal amplification of a DNA region. **(C)** Break-Induced Replication (BIR), which participates in alternative telomere lengthening as well as in expansion of certain short tandem repeats. Gene copies are shown in orange. The sequence of the origin of replication, which is incorporated into the extrachromosomal circular DNA, is shown in purple. Red dotted lines indicate sites of non-allelic homologous recombination. RNA in RNA-DNA hybrid is shown in green. The purple circles indicate regions of double-strand breaks. Key proteins in each pathway are indicated in red. Schemes were created using BioRender. Abdurazakov, A. (2025).

As mentioned above, a close association between R-loops and HR has been demonstrated, and HR is one of the main causes of both intra- and extrachromosomal DNA amplifications. An increase in the DNA copy number can occur if recombination takes place between homologous sequences located in non-allelic positions, i.e., between identical sequences that are not localized at the same loci of homologous chromosomes. Such regions may reside on the same chromosome (intrachromosomal recombination), or on a different loci of the homologous chromosome (unequal exchange between sister chromatids) (Hastings et al., 2009). Intrachromosomal HR results in excision of a DNA fragment. Ligation of the ends of such a fragment may lead to the formation of an eccDNA molecule (Figure 2A). If the resulting circular DNA contains an origin of replication, its copy number may subsequently increase through autonomous replication (Gresham et al., 2010). On the other hand, interchromosomal HR can lead to an increase in the number of DNA copies within the chromosomes (Figure 2B) (Hastings et al., 2009). Non-Allelic HR (NAHR) is frequently observed in repetitive genomic regions. A well-characterized example is ribosomal DNA, which is organized as clusters of tandem repeats. Unequal exchange between sister chromatids has been shown to result in ribosomal genes amplification, while, intrachromosomal recombination within a ribosomal gene cluster leads to the formation of extrachromosomal ribosomal circles (ERCs) (Kobayashi and Ganley, 2005).

Importantly, HR variant known as Break-Induced Replication (BIR) is frequently associated with R-loop-mediated replication fork stalling (Figure 2C) (Costantino et al., 2014). During this process after the initial 5′-terminal DNA shortening (resection), characteristic for any type of HR, an invasion of the single-stranded DNA region into the homologous region of the donor chromosome occurs leading to the formation of a new replication fork. If the homologous (donor) region is located in a non-allelic position, duplication of the corresponding DNA fragment will happen. The best-described example of BIR is the increase in the copy number of short repeating motifs (Kim et al., 2017) (see section 6.3).

In some cases, BIR can occur during mitosis. This specialized process is known as Mitotic DNA Synthesis (MiDAS) (reviewed by Bhowmick et al., 2023). The MiDAS machinery is characterized by the presence of specific protein components. For example, whereas BIR (like classical HR) involves the BLM helicase, MiDAS instead utilizes the WRN (Barwacz et al., 2025) and RTEL helicases, with the latter playing an important role in G4-quadruplexes unwinding (Wu et al., 2020). MiDAS frequently occurs at so-called fragile sites–genomic regions with an increased propensity to form DSBs. These regions typically comprise long (300 kb or more) transcribed genes or loci with complex secondary structures (telomeres, short tandem repeats) (Wu et al., 2020; Khristich and Mirkin, 2020). Fragile sites often remain underreplicated at the onset of cell division, and in such cases, their replication is completed via the MiDAS pathway during mitosis. Therefore, MiDAS can be activated even under normal physiological conditions. However, factors that promote replication fork stalling - for example, low doses of DNA-polymerase inhibitor Aphidicolin or folate deficiency–have been shown to substantially upregulate MiDAS frequency, leading to genome instability (Wu et al., 2020; Garribba et al., 2020; Groelly et al., 2022; Bhowmick et al., 2023). MiDAS pathway participates in telomerase-independent telomere elongation (ALT) (Min et al., 2017) (see Section 6.2), and also contributes to the expansion of certain tandem repeats (see Section 6.3). R-loops at fragile site regions have been shown to recruit the FANCD2 protein, which attracts other proteins involved in MiDAS, including the RTEL1 helicase and the POLD3 DNA polymerase (Wu et al., 2020). Consistent with this, Wu et al. demonstrated that overexpression of RNase H1 decreased MiDAS frequency (Wu et al., 2020). Therefore, multiple lines of evidence point to the involvement of R-loops in the initiation of MiDAS-mediated DNA copy number amplification.

### 5.2 Microhomology-based mechanisms

This group of pathways includes RAD51-independent mechanisms such as Microhomology-Mediated BIR (MMBIR) and Fork Stalling and Template Switching (FoSTeS). MMBIR is generally similar to classical BIR. It enables the cell to repair DSBs that result from replication fork collapse (So et al., 2017). However, MMBIR is activated when the single-stranded 3′DNA end of a damaged chromosome cannot anneal to a homologous sequence on the donor chromosome. It may occur due to a deficiency of the RAD51 protein, which plays a key role in a screening for homologous DNA regions and mediates the subsequent invasion of the single-stranded 3′DNA end (Hastings et al., 2009). As a result, the 3′terminus hybridizes with any available region of the donor chromosome containing a 5–25 bp long microhomology site (Truong et al., 2013). After a short elongation phase, the newly synthesized DNA fragment dissociates and anneals to a new template. Multiple rounds of elongations on different templates may eventually lead to rehybridization of the extended 3′terminus with a region near the initial replication fork, thereby restoring DNA synthesis to its initial template. These template-switching events can introduce substantial genomic rearrangements, including amplification of donor chromosome segments (So et al., 2017).

**FIGURE 3:**
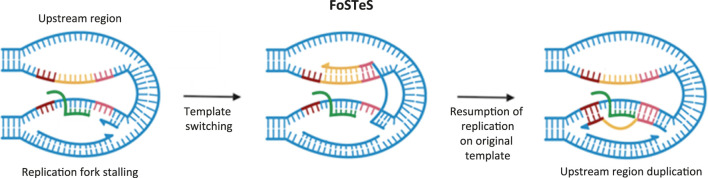
Schematic representation of the fork stalling and template switching (FoSTeS) mechanism. The duplicated upstream region is shown in orange; microhomology regions are shown in brown and pink; RNA molecule is shown in green. Schema was created using BioRender. Abdurazakov, A. (2025).

On the other hand, FoSTeS is initiated by replication fork stalling (for example, at a site of R-loop) and does not involve the formation of DSBs and 5′-end resection, which are characteristic of HR and MMBIR. During this mechanism, one of the newly synthesized DNA strands anneals to another genomic region that contains single-stranded DNA with a microhomology site. Subsequent DNA elongation, similar to MMBIR, can lead to duplication of a fragment of the donor chromosome (Zhang et al., 2009) (Figure 3).

Both MMBIR and FoSTeS are sources of DNA duplications observed in patients with neurofibromatosis type 1 (Hsiao et al., 2015). These mechanisms are also activated in cancer cells deficient in RAD51 (Hastings et al., 2009). Although, a direct link between these pathways and R-loops has not yet been established, it has been shown that MMBIR participates in the repair of DSBs caused by replicative stress, while FoSTeS facilitates the bypass of replication fork arrest (So et al., 2017; Zhang et al., 2009). Since both replicative stress and replication arrest can be triggered by R-loops, a connection of R-loops with MMBIR and FoSTeS appears highly probable.

### 5.3 NHEJ-based mechanisms

Non-Homologous End Joining (NHEJ)-mediated group of pathways includes Breakage-Fusion-Bridge cycles (BFB-cycles) and chromothripsis–mechanisms that induce large-scale genome rearrangements. NHEJ enables cells directly ligate DNA ends even in the absence of homologous sequences. The Ku80-Ku70 protein complex plays a critical role in this process (So et al., 2017). Several studies have shown that R-loops may contribute to the initiation of various NHEJ pathways (Stork et al., 2016; Tang et al., 2022; Lee et al., 2023).

**FIGURE 4:**
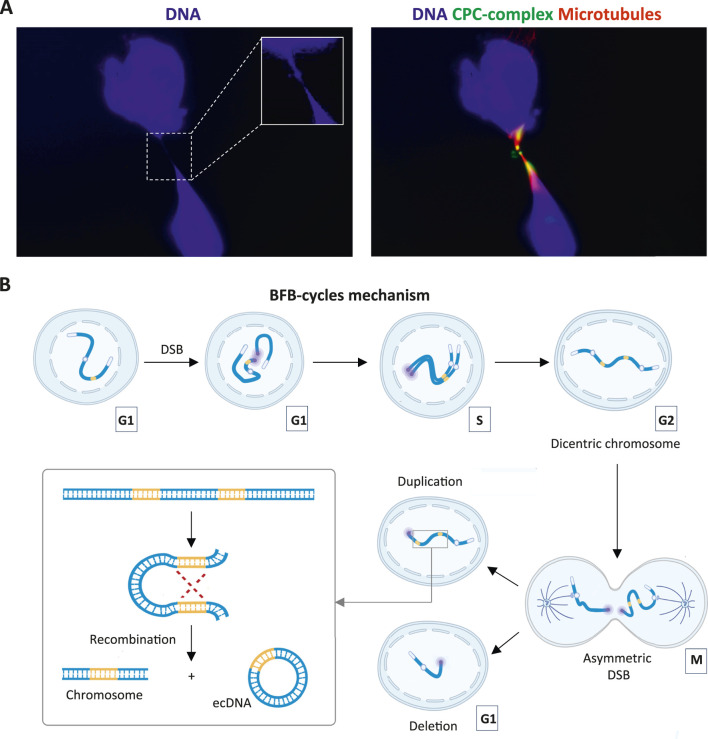
Breakage-Fusion-Bridge (BFB) cycles model. **(A)** Immunofluorescent image showing a chromosomal bridge formed by dividing fibrosarcoma cell. Cells were stained with antibodies against Chromosomal Passenger Complex (CPC) protein Survivin (green), acetylated α-Tubulin (red) and DNA stain DAPI (blue). **(B)** Schema illustrating intra- and extrachromosomal amplification via the BFB-cycles mechanism. Gene copies are shown in orange. Red dotted lines indicate sites of homologous recombination. Purple circles show sites of double-strand breaks. White ovals denote telomeric regions. White circles show centromeres. The schema was created using BioRender. Abdurazakov, A. (2025), the microscopic images were obtained by the authors.

In 1953, B. McClintock proposed BFB-cycles gene amplification model (McClintock, 1951). This process begins with the loss of a telomere and formation of an unprotected chromosomal end. Telomere loss can occur due to a DSB, a telomeric crisis (loss of telomeres due to their shortening) or chromosome underreplication (Shimizu et al., 2005). After replication of a chromosome lacking a telomere, two sister chromatids without telomeres are formed. These chromatids are next joined at the DSB site via NHEJ, producing a dicentric chromosome. During anaphase of the subsequent mitosis, the centromeres of the dicentric chromosome are pulled toward opposite poles of the cell, creating a chromosomal bridge at the middle (Figure 4A). The bridge breaks at a random location. As a result, one daughter cell receives a chromosome containing duplicated genes, while the other daughter cell acquires a chromosome lacking those genes (Zong et al., 2012). The BFB cycle can then be repeated multiple times, leading in further amplification of the affected chromosomal region (Figure 4B). Subsequent HR between duplicated gene copies may give rise to extrachromosomal DNA (ecDNA) (Tanaka and Yao, 2009). Therefore, BFB-cycles can promote both intra- and extrachromosomal amplification of genomic regions. An example of this mechanism is the amplification of the *DHFR* gene, which can be induced in cancer cells by the DHFR inhibitor Methotrexate. This compound has been shown to promote formation of ecDNA encoding DHFR as well as intrachromosomal amplification of the corresponding gene (Shoshani et al., 2021).

Although a direct interconnection between R-loops and the classical BFB mechanism has not been demonstrated, a recent study showed that RNA-DNA hybrids can promote translocation-bridge amplification, a process mechanistically similar to BFB cycles. Translocation-bridge amplification occurs when DSBs arise simultaneously in two different chromosomes, for example, as a result of replicative stress. Subsequent ligation of fragments from different chromosomes leads to the formation of a dicentric fusion chromosome. In contrast to the classical BFB mechanism, this ligation occurs prior to DNA replication. Breakage of the resulting chromosomal bridge during mitosis can induce subsequent BFB cycles, leading to interchromosomal translocations, co-amplifications, and the formation of ecDNA, which in this case will contain genes originally located on different chromosomes (Lee et al., 2023). Importantly, a direct association between translocation-bridge amplification mechanism and R-loops has been demonstrated. Stork et al. showed that in breast cancer cells, R-loops formed in the estrogen receptor target genes enhance DSBs formation (Stork et al., 2016). As was later demonstrated by Lee et al., such DSBs serve as triggers for translocation-bridge amplification, which induces amplification of the corresponding genes (Lee et al., 2023) (see section 7).

Another (NHEJ)-mediated mechanism of gene amplification is Chromothripsis. Chromothripsis is a catastrophic genomic event in which one or more chromosomes undergo extensive fragmentation, and the resulting DNA fragments are rejoined via NHEJ often in an incorrect order (reviewed in Ly and Cleveland, 2017). Some of these fragments may be lost and will not participate in the formation of the reassembled chromosome, while others can be ligated to form large circular DNA molecules (ecDNAs), which may be further amplified during subsequent DNA replication rounds.

Chromothripsis has been identified in many malignancies, including lung, prostate and pancreatic cancer, as well as neuroblastoma (George et al., 2015; Fraser et al., 2017; Notta et al., 2016; Molenaar et al., 2012). A variety of factors can induce chromothripsis, including abnormalities in chromosome segregation during mitosis. It has been shown that the “lagging” chromosomes, which enter the daughter cell after nuclear envelope formation, are subject to chromothripsis (Ly and Cleveland, 2017). These chromosomes become encapsulated in micronuclei - small nucleus-like structures surrounded by their own nuclear membrane (Fenech et al., 2011). Tang et al. reported increased R-loops formation on chromosomes in micronuclei. The authors demonstrated that DNA within these R-loops undergoes extensive ADAR-dependent editing, leading to subsequent formation of numerous DSBs (see section 2). Thus, R-loops in micronuclei promote DNA fragmentation and contribute to the induction of chromothripsis (Tang et al., 2022).

## 6 The role of R-loops in the amplification of repetitive genome sequences

As described above, R-loops are involved in many DNA amplification mechanisms. Below, we present specific examples of intra- and extrachromosomal amplifications that are known to be initiated by these structures.

### 6.1 The role of R-loops in copy number variations of ribosomal DNA and the formation of extrachromosomal ribosomal circular DNA

The most compelling evidence for the involvement of R-loops in the amplification of ribosomal RNA (rRNA) genes has been obtained in yeast. In *Saccharomyces cerevisiae*, rRNA genes are located on chromosome XII in approximately 150 copies (Petes, 1979). In a series of studies, Kobayashi et al. investigated the mechanism of yeast ribosomal DNA (rDNA) amplification, which is triggered when the copy number of rDNA falls below a critical threshold. The authors demonstrated that this amplification is triggered by HR events which repair DSBs. These DSBs arise as a result of replication fork stalling (Kobayashi et al., 2004), that, in turn, is initiated by the transcription of long non-coding RNAs (lncRNAs) originating from the intergenic spacers within the ribosomal gene cluster. These lncRNAs form R-loops on the rDNA sequences (Kobayashi and Ganley, 2005; Saka et al., 2013). Non-allelic recombination between sister chromatids leads to intrachromosomal amplification of rDNA. In addition, recombination can also occur within the same ribosomal gene cluster, resulting in the formation of circular DNA molecules containing rDNA—extrachromosomal ribosomal circles (ERCs) (Kobayashi and Ganley, 2005).

The mechanism described above may also exist in human rDNA loci. The copy number of human rDNA varies greatly among individuals, ranging from 67 to 412 copies per cell (Gibbons et al., 2014). In addition, rDNA instability has been observed in cancer. Valori et al. found both increases and decreases in rDNA copy number in breast cancer cells compared to normal tissue (Valori et al., 2020). eccDNAs containing rDNA sequence have also been identified in humans (Møller et al., 2018). It is important to note, that similarly to yeast, various non-coding transcripts are synthesized from human rDNA, and some of these can form R-loops (Abraham et al., 2020). Therefore, it is likely that R-loops may contribute of rDNA copy number variation in humans. However, the direct association between R-loops and rDNA amplification in human cells has not yet been investigated.

### 6.2 Involvement of R-loops in alternative telomere lengthening and formation of C- and T-circles

Although telomeric DNA regions exhibit heterochromatic characteristics, they are actively transcribed to produce a group of lncRNAs known as telomeric repeat-containing RNA–TERRA (Barral and Déjardin, 2020; Feretzaki et al., 2019). TERRA lncRNAs have been shown to form R-loops at telomeres. In this case, RNA-DNA hybrid formation occurs post-transcriptionally *in trans.* It is facilitated by RAD51 protein, which recruits TERRA to telomeric sequences (Feretzaki et al., 2020). Under certain conditions, these R-loops can promote replication stress and DSBs formation. Repair of these breaks through HR-mediated mechanisms increases telomeric repeats copy number. There is evidence indicating that this process may also occur during mitosis via the MiDAS pathway (Min et al., 2017; Epum and Haber, 2022). Therefore, telomeric R-loops promote telomere lengthening via the telomerase-independent ALT mechanism (see Section 4). In line with this observation, Silva et al. demonstrated that inhibition of TERRA transcription in osteosarcoma cells reduced replicative stress and telomere DNA damage, ultimately preventing telomere elongation (Silva et al., 2021). A similar effect was observed when RNase H1, an endonuclease that cleaves RNA in RNA-DNA hybrids, was overexpressed (Arora et al., 2014).

One of the consequences of ALT activation is the formation of telomeric repeats-containing circular DNAs, which include single-stranded C-circles and double-stranded T-circles. These structures are capable of autonomous replication via a rolling-circle mechanism (reviewed in Hou et al., 2022). There are evidences indicating that these extrachromosomal repeats can be reintegrated into the telomeric regions of chromosomes. Thus, extrachromosomal telomeric DNA may contribute to telomerase-independent telomere maintenance (Nosek et al., 2005). Work of Poole and colleagues showed that the reduction of the levels of SMARCAL1 protein - a DNA translocase involved in replication fork restart following replication stress - in cervical cancer cells and osteosarcoma cells increases the amount of extrachromosomal circular telomeric DNA (Poole et al., 2015). Furthermore, decreased levels of the ATPase/translocase FANCM, which unwinds DNA-RNA hybrids, have been shown to elevate the amount of C-circles (Silva et al., 2019). Together these findings suggest that telomeric R-loops may contribute to the formation of extrachromosomal telomeric circular DNAs.

### 6.3 R-loops and short tandem repeats expansion

Short Tandem Repeats (STRs), or microsatellites, are genomic elements composed of repetitive motifs of 2–12 bp in length (Malik et al., 2021). These elements are widely present in the human genome. Over the past 2 decades, it has become clear that STRs perform important functions in the cell (reviewed by Bagshaw, 2017). These structures can influence gene expression at both the transcriptional and post-transcriptional levels. Thus, STRs located in promoter regions can bind various transcription factors. Due to their structural flexibility, some STRs form loops that facilitate spatial proximity between regulatory elements such as enhancers and promoters. In certain cases, STR length polymorphism can affect transcription initiation and termination sites selection, as well as the usage of RNA splice sites. STRs have also been shown to influence chromatin organization and transcript stability (Bagshaw, 2017). Extensive STR length polymorphism (microsatellite instability) has been observed in cancers (Nojadeh et al., 2018; Cortes-Ciriano et al., 2017). Moreover, a significant increase in the copy number of short repetitive motifs - referred to as repeat expansion - is associated with many neurodegenerative diseases, including fragile X-chromosome syndrome, myotonic dystrophy types 1 and 2, spinocerebellar ataxia, and Fredreich’s ataxia (Malik et al., 2021; Bagshaw, 2017).

Different repeats undergo expansion via different molecular mechanisms (reviewed in Khristich and Mirkin, 2020). Tandem repeat expansion can occur both during DNA replication, due to DNA strand misalignment, and during transcription, where R-loops may initiate the process. Although, replication-associated mechanisms of STR instability are beyond the scope of this review, it is interesting to note that the pathway responsible for repeat expansion—replication- or transcription-mediated—primarily depends on the nucleotide sequence of the STR and for some STRs, the role for R-loops in their expansion has been clearly demonstrated. Thus, it has been shown that stable R-loops are formed on G-rich repeats (e.g., GAA, GGGGCC, CGG, CAG), as the single stranded DNA in these regions tends to form secondary structures (loops, G4-quadruplexes) that stabilize R-loops (Groh et al., 2014; Loomis et al., 2014). Consistent with this, Neil et al. demonstrated that R-loops contribute to the expansion of the (GAA)n repeat in Friedreich’s ataxia (Neil et al., 2018). Furthermore, RNase H1 has been shown to prevent CAG repeat instability in mammalian cells (Lin et al., 2010). Summing up, it can be proposed that the formation of R-loops induces DSBs, whose repair through HR can alter the copy number of the corresponding tandem repeat. Indeed, multiple studies have shown that the expansion of CAG, GAA and CGG repeats is associated with BIR or MiDAS (see section 5.1) (Neil et al., 2018; Kim et al., 2017; Kononenko et al., 2018; Hayward and Usdin, 2021).

## 7 The role of R-loops in the amplification of unique sequences

As described above, the role of R-loops in the amplification of repetitive genomic regions (ribosomal and telomeric DNA, short tandem repeats) has been demonstrated in numerous studies. As opposed to this, the involvement of R-loops in the amplification of unique genomic sequences remains less clear. Nonetheless, several examples of gene amplification events in which R-loops are likely to participate have been described in the literature. Thus, Lee et al. found that incubation of breast cancer cells with estrogen led to the formation of DSBs near estrogen receptor target genes, followed by the amplification of the corresponding sequences, which included *CCND1* and *ZNF703* (Lee et al., 2023). The authors showed that amplification of these genes occurred via a translocation–bridge mechanism. These findings are in a good agreement with earlier work by Stork et al., who demonstrated that incubation of MCF-7 breast cancer cells with estrogen increases transcriptional activity, as well as the levels of R-loops and DSBs (Stork et al., 2016). Importantly, RNase H1 overexpression significantly reduced the amount of DSBs, indicating the involvement of estrogen-induced R-loops in the initiation of DNA damage. Mapping of these R-loops revealed that they were predominantly associated with estrogen receptor target genes, including the aforementioned *CCND1* and *ZNF703*. Based on the above results, it can be concluded that estrogen-induced increase in transcription may lead to R-loops accumulation and the formation of DSBs near estrogen receptor targets. This, in turn, may theoretically causes genomic translocations and subsequent intrachromosomal amplifications in cancer cells.

## 8 Discussion

Gene amplification is a hallmark of cancer cells that contributes to increased tumor malignancy and the development of the therapy resistance. Understanding the mechanisms underlying both intra- and extrachromosomal amplification is therefore of critical importance. To date, the prevailing view in the field is that, in cancer cells, amplification of DNA regions occurs randomly, and clones that acquired additional copies of beneficial genes gradually outcompete those with less advantageous alterations (Greaves and Maley, 2012). However, a growing body of evidence suggests that the formation of R-loops may facilitate the selective amplification of highly expressed genes. This process could enable tumor cells to further enhance the expression of key oncogenes, thereby increasing their adaptive capacity and malignant potential.

Multiple mechanisms contribute to the accumulation of R-loops in cancer cells. First, mutations or downregulation of splicing factors and RNA-DNA helicases, such as DDX41, have been shown to promote R-loops formation (Arif et al., 2023; Mosler et al., 2021). On the other hand, accumulation of R-loops in specific regions of the genome can be promoted by elevated transcriptional activity of the corresponding genes in cancer cells. One example is the enhanced R-loops formation near estrogen receptor target genes (see Section 7). Finally, defects in components of the DNA repair machinery, such as BRCA1 and BRCA2, can facilitate persistence of unresolved DNA-RNA hybrids (Hatchi et al., 2015; Gondo et al., 2021). Collectively, these factors contribute to R-loop accumulation in malignant cells, which, in turn, may promote further genomic instability and gene amplification.

R-loops have been shown to contribute to the increase in the copy number of repetitive genomic elements (ribosomal RNA genes, telomeric and short tandem repeats), as well as certain unique sequences. It is also plausible that R-loops are involved in the generation of oncogene-containing ecDNA molecules, which are frequently observed across diverse cancer types (Kim et al., 2024). Notably, different malignancies exhibit amplification of distinct sets of genes. For example, endometrial cancer is characterized by intrachromosomal amplification of *ESR1, KRAS*, *PIK3CA, ERBB2, TERC, MYC, CCNE1* (Rahman et al., 2013; Birkeland et al., 2012; Konopka et al., 2011; Cherniack et al., 2017). In neuroblastoma, ecDNA promotes high level amplification and extensive intratumoral heterogeneity of the MYCN oncogene, which is associated with poor clinical outcomes (Koche et al., 2020; Montuori et al., 2025). In glioblastoma both intra- and extrachromosomal amplification of *EGFR, PDGFRA, MET, MECOM/PIK3CA/SOX2* gene cluster and *CDK4/MDM2* are frequently observed (deCarvalho et al., 2018). Lastly, esophageal cancer often exhibits intrachromosomal amplification of *MYC*, *ERBB2, EGFR, RB1, GATA4/6, CCND1, RTK* and *MDM2* as well as ecDNA-associated amplification of *MYC* and *MDM2* (Testa et al., 2017). One potential explanation for such cancer-type-specific patterns of oncogene amplification is that they might be initiated by transcriptional upregulation of the corresponding genome regions. This upregulation could promote R-loops formation and increase susceptibility to DSBs, thereby facilitating subsequent gene amplification. However, further research is required to test this hypothesis.

## 9 Conclusion

Oncogene amplification—both intrachromosomal and in the form of extrachromosomal circular DNA—is a major driver of cancer progression and therapy resistance. Numerous studies have highlighted the critical role of such amplification events in enabling cancer cells to evade targeted therapies (Morales et al., 2009; Shoshani et al., 2021). Inhibition of these pathways may represent a promising strategy to suppress the emergence of drug resistance and improve patient outcomes.

While the molecular mechanisms of CNV formations have been extensively studied, the initial triggers of these processes remain poorly understood. In this review, we described examples of R-loop-initiated gene amplification, including those implicated in cancer. However, further research is needed to clarify the contribution of R-loops in genome rearrangements in malignant cells. Particularly intriguing is the potential link between R-loops and ecDNAs which have recently been shown to play a critical role in cancer progression.
